# Impact of Maternal Environment and Inflammation on Fetal Neurodevelopment

**DOI:** 10.3390/antiox13040453

**Published:** 2024-04-11

**Authors:** Chiara Lubrano, Francesca Parisi, Irene Cetin

**Affiliations:** 1 Nutritional Sciences, Doctoral Programme (PhD), Università degli Studi di Milano, 20157 Milan, Italy; chiara.lubrano@unimi.it; 2 Department of Mother, Child and Neonate, Fondazione IRCCS Ca’ Granda Ospedale Maggiore Policlinico, 20122 Milan, Italy; 3 Department of Biomedical and Clinical Sciences, Università degli Studi di Milano, 20157 Milan, Italy; irene.cetin@unimi.it

**Keywords:** fetal neurodevelopment, maternal nutrition, anxiety, depression, air pollution, socioeconomic status, inflammation, nutrient supply, oxidative stress

## Abstract

During intrauterine life, external stimuli including maternal nutrition, lifestyle, socioeconomic conditions, anxiety, stress, and air pollution can significantly impact fetal development. The human brain structures begin to form in the early weeks of gestation and continue to grow and mature throughout pregnancy. This review aims to assess, based on the latest research, the impact of environmental factors on fetal and neonatal brain development, showing that oxidative stress and inflammation are implied as a common factor for most of the stressors. Environmental insults can induce a maternal inflammatory state and modify nutrient supply to the fetus, possibly through epigenetic mechanisms, leading to significant consequences for brain morphogenesis and neurological outcomes. These risk factors are often synergic and mutually reinforcing. Fetal growth restriction and preterm birth represent paradigms of intrauterine reduced nutrient supply and inflammation, respectively. These mechanisms can lead to an increase in free radicals and, consequently, oxidative stress, with well-known adverse effects on the offspring’s neurodevelopment. Therefore, a healthy intrauterine environment is a critical factor in supporting normal fetal brain development. Hence, healthcare professionals and clinicians should implement effective interventions to prevent and reduce modifiable risk factors associated with an increased inflammatory state and decreased nutrient supply during pregnancy.

## 1. Introduction

Human brain development begins around the second–third gestational week and extends through childhood, with a complex interplay of genetics, epigenetics, and environment factors influencing both short- and long-term outcomes [1]. The period between the 8th and 18th gestational weeks plays a pivotal role in the development of the neocortex, which is responsible for sensation, action, cognition, and consciousness. During this time window, extensive neurogenesis and cellular migration occur, wherein Neural Progenitor Cells (NPCs) undergo division and differentiation into neurons and glia, the two fundamental cell types of the nervous system. The differentiation of brain structures occurs around mid-pregnancy, along with the development of the cerebellum and the onset of myelination. During the third trimester, a rapid brain growth, particularly in the cerebral cortex, with increased myelination, further maturation of sensory organs, and refinement of neural connections, occurs [2]. Finally, the blood–brain barrier is established [3]. Any derangement of these developmental processes may lead to severe short- and long-term consequences.

Before and during pregnancy, maternal environmental exposures are known to impact not only on short-term intrauterine development, but also on long-term offspring overall health, increasing the risk of non-communicable diseases during adulthood, such as cancer, heart disease, obesity, or diabetes. Prenatal exposures to external stimuli may lead to modifications in fetal and placental phenotype through epigenetic mechanisms, resulting in the “reprogramming” of organ structure, physiology, and metabolism [4,5,6,7]. Organs with greater plasticity, like the brain, are more susceptible to these effects. Although the significant role of the prenatal environment in determining the adult phenotype is more and more supported, the molecular mechanisms underlying the associations between external factors and neurodevelopment alterations are poorly understood.

Several human studies investigated the associations between the external maternal environment and offspring neurodevelopment by using infant questionnaires and tests, while a few studies explored the association with fetal brain morphology and growth by utilizing ultrasound or Magnetic Resonance Imaging (MRI) during pregnancy. Nevertheless, as the brain’s development is a dynamic and multifaced process influenced by both genetic, environmental, and experiential factors, assessing the real causative effect of a single exposure on brain development is often challenging. Additionally, external stimuli often act synergically. Therefore, we included animal studies as experimental models providing potential causal explanations for the pathophysiological pathways linking single maternal exposures to fetal neurodevelopment.

The present review aims to examine the available evidence on the impact of single maternal environmental exposures, diet, and lifestyle on intrauterine brain development and child neurodevelopmental outcome. An extensive literature search was performed using Pubmed and Scopus with the following terms: “maternal nutrition” [All Fields] OR “Mediterranean diet” [All Fields] OR “iodine” [All Fields] OR “docosahexaenoic acid (DHA)” [All Fields] OR “Iron” [All Fields] OR “anemia” [All Fields] OR “stress” [All Fields] OR “Depression” [All Fields] OR “Anxiety” [All Fields] OR “Alcohol” [All Fields] OR “Smoke” [All Fields] OR “Air pollution” [All Fields] OR “Socioeconomic status” [All Fields] OR “Inflammation” [All Fields] AND “fetal development” [All Fields]) OR (“fetal disease” [All Fields] OR “neurodevelopmental disorders” [All Fields] OR “brain alterations” [All Fields] OR “neurodevelopmental” [All Fields]. Additionally, we conducted a manual search to obtain articles listed in the reference lists of articles found in the initial search. The search encompassed observational, retrospective, and prospective studies, as well as case–control, cohort studies, systematic reviews, and meta-analyses. It was limited to English-language studies published within the last 15 years (2009–2024). The review included all articles providing sufficient information on the association between external stimuli and fetal neurodevelopment, including both human and animal studies.

## 2. Environment, Lifestyle, and Specific Maternal Phenotypes and Risk Factors

Table 1 summarizes the primary human studies assessing the association between external stimuli and fetal brain abnormalities. A detailed description of the main maternal environmental stressors potentially affecting intrauterine neurogenesis is provided in the following paragraphs.

Table 2 summarizes the available animal studies on the effects of external factors on the offspring’s neurodevelopment. Animal models provide the opportunity to control the type, intensity, duration, and timing of maternal stressors, as well as to observe the interaction of the mother with her offspring in a controlled model. Furthermore, pregnancy in animals has a shorter duration than in humans (rats = 21.5 days and monkeys = 165 days vs. human = 270 days), allowing researchers to investigate the long-term outcomes in a shorter time frame.

### 2.1. Maternal Nutrition

The 1944 Dutch famine represents one of the first settings providing evidence of an association between maternal nutritional environment and neurodevelopment outcome in the offspring, firstly, by showing increased rates of congenital abnormalities of the central nervous system [46]. Indeed, it has been observed that insufficient maternal nutrient intake during the early stages of pregnancy impacts neural cell proliferation, while in the later stages, mainly affects neural differentiation [47]. Mouse models confirmed that a low-protein diet affects fetal neurodevelopment, resulting in delays in surface righting reflex and negative geotaxis response [20]. This effect may be explained by the reduction in expression of BDNF (Brain-Derived Neurotrophic Factor) in the hypothalamus and motor cortex [21]. BDNF is a neurotrophic protein that plays a crucial role in the development, survival, and plasticity of neurons. Additionally, in baboon models, fetuses of undernourished mothers exhibited suppression of neurotrophic factor, imbalanced cell proliferation, and impaired glial maturation and neural synthesis [22,46]. Alterations in the methylation process are strongly associated with maternal undernutrition and alterations in the activity of the fetal hypothalamic–pituitary–adrenal axis, leading to changes in cortisol levels [47,48], which could contribute to an increased risk of schizophrenia, antisocial personality disorder, and admittance to addiction programs. These behaviors are more frequent in fetuses born to undernourished mothers [49]. In line with these results, unhealthy dietary patterns, such as the Western one characterized by low-quality and low-cost foods, result in excessive intake of macronutrients, which is concomitant with micronutrient deficiencies. Therefore, the fetal brain may experience both a lack of crucial components, such as DHA, folate, or iodine, and excessive exposure to macronutrients (i.e., carbohydrates and fats). The Mediterranean diet has been considered one of the healthiest dietary patterns in the world. Several studies investigating the associations between the Mediterranean diet and brain health in the adult population show larger brain volume, higher executive function scores, improved white matter integrity, and reduced risks of Alzheimer’s and Parkinson’s diseases in adults highly adherent to this dietary pattern [50,51,52]. Similarly, maternal adherence to the Mediterranean diet during periconception and pregnancy improved social relatedness behaviors and autonomic stability, reducing depression, anxiety, atypical behaviors, and the risk of autism spectrum disorders in the infant population [8,53].

Nevertheless, few studies assessed fetal neurodevelopment in relation to the maternal diet. A significant association was reported in a randomized controlled trial involving 90 pregnant women between maternal Mediterranean diet and larger total fetal brain volume, corpus callosum, and right frontal lobe, with higher scores being recorded for autonomic stability at 1–3 months of life [8]. Other maternal dietary patterns, such as ketogenic diet, which is characterized by low carbohydrates and high fat, may impact on fetal neurodevelopment [54]. Animal models demonstrated an association between the ketogenic diet and reduced glucose uptake in the brains of offspring, leading to the larger volume of the cerebellum and spinal cord, as well as reductions in the volume of the cerebral cortex, hippocampus, corpus callosum, and lateral brain ventricles [55,56,57]. Indeed, ketones are unable to replace the critical function of glucose in prenatal neurodevelopment [58]. This subsequently resulted in the development of hyperactivity and anxiety in adult offspring [47].

However, the role of specific aspects of nutrition and micronutrients in determining fetal neurodevelopment is not completely understood [1].

DHA, a n-3 long-chain polyunsaturated fatty acid (LCPUFA), regulates the functions of synaptic proteins and the membranes of astrocytes, microglia, and oligodendrocytes. Animal models showed that DHA supplementation during pregnancy was associated with a significant increase in the number of hippocampal neural cells [23]. Indeed, its accumulation in the fetal brain during the third trimester is crucial for fetal neurodevelopment [59,60,61]. Ogundipe et al. identified a significant effect of supplementation, with 600 mg DHA taken during pregnancy for at least 13 weeks impacting on larger head circumference at birth, as well as on larger total brain, cortex, whole gray matter, and corpus callosum volumes compared to the placebo group, and, additionally, observed higher neurodevelopmental scores at two years [9]. Animal models showed that insufficient intake of n-3 fatty acids resulted in reduced DHA levels in the brain, leading to impaired neurogenesis, altered neurotransmitter metabolism (dopamine and serotonin), and compromised learning and visual function [24,62]. In contrast, excessive exposure to PUFAs during pregnancy may lead to an altered stress response and a tendency to avoid open spaces. This behavior, known as thigmotactic behavior, is considered an index of anxiety in the offspring [47,63]. Further human studies confirmed the association between DHA supplementation and improved development and cognitive outcomes in children [59,64,65].

Iodine deficiency is one of the most common micronutrient deficiencies worldwide and a recognized cause of preventable impairment of mental function [1]. Iodine plays a pivotal role in the production of thyroid hormones, which regulate neurogenesis, promote neuronal maturation, and facilitate myelination. Additionally, thyroid hormones also act as transcription factors, regulating the expression of genes involved in brain development [26]. Animal studies observed that maternal hypothyroidism during pregnancy is associated with a reduction in the CREB (cAMP response element-binding protein) pathway and BDNF protein expression [25,26]. CREB is a nuclear transcription factor that plays a crucial role in many aspects of neuronal development, including the survival and proliferation of neurons, synapse formation, neuronal synaptic plasticity, and long-term memory formation. A reduction in the CREB pathway resulted in irreversible neurodevelopmental damage in rat pups, which could not be recovered even with prolonged training [26]. A reduction in BDNF expression is observed in the developing hippocampus of pups from both subclinical and hypothyroid mothers, which is associated with deficits in both short-term and long-term spatial memory [25]. Considering this, it is not surprising to find an association between severe maternal iodine deficiency and neurodevelopmental issues in human offspring, including motor function deficits, cognitive impairment, language delay, behavioral disorders, and hypodevelopment [66,67]. However, iodine supplementation is recommended in areas with severe iodine deficiency (Urinary Iodine Concentration (UIC) < 50 μg/L), whereas its efficacy is not well supported by quality evidence in regions with mild-to-moderate iodine deficiency (UIC 50–150 µg/L). Indeed, in women with a habitually low iodine intake (<160 μg/d), iodine supplementation is negatively associated with child behavior, including an increased risk of attention deficit hyperactivity disorder (ADHD) and internalizing-behavior problems [68,69].

Iron deficiency (ID) anemia (IDA) is the most common nutritional deficiency among pregnant women, with a reported prevalence as high as 15–20% [70]. Iron is an essential trace element for myelinization promotion, hemoglobin formation, and oxygen delivery to the developing brain [1,71]. Most symptoms observed in individuals with IDA are associated with a lack of neuronal iron [71]. The impact of maternal ID on the fetal brain depends on the affected brain region and the gestational week in which it occurs, potentially leading to permanent structural deficits [72,73]. Additionally, fetal ID increases the postnatal risk of long-term mental health abnormalities such as autism, schizophrenia, and neurocognitive disorders [1,74]. Indeed, animal models showed that alterations in the neurodevelopment of rat pups with maternal ID were associated with significant epigenetic modifications in hippocampus and cerebellum, resulting in significant short- and long-term reprogramming of gene expression. Specifically, alterations in DNA methylation, DNA hydroxymethylation, and histone methylation due to maternal ID were associated with persistent downregulation of BDNF, resulting in long-lasting impairments in cognition and socio-emotional behaviors [27,28,29].

Folic acid is required for neural cell proliferation, migration, differentiation, vesicular transport, and synaptic plasticity, and its role in preventing neural tube defects and neurodevelopmental disorders is widely accepted [47,75]. During pregnancy, the demand for folic acid increases by around 50%, leading to the recommendation of supplementation of 400–800 μg for all women of childbearing age from two months before to three months after conception [76,77]. Animal models have shown that maternal folic acid deficiency reduces neuronal cell proliferation by impairing mitosis and increases apoptosis [30]. Folate, serving as an important methyl donor, influences DNA and histone methylation, leading to the downregulation of neural gene expression and impaired fetal neurogenesis [31]. Additionally, folic acid supplementation reduces the risk of autism spectrum disorders (ASD) in offspring by nearly 40% [78,79,80]. However, two studies have shown that higher supplementation (≥1000 μg) was associated with a higher risk of ASD [81,82]. Overall, moderate amounts of folic acid supplementation may be effective in the prevention of ASD [83].

Vitamin D is a steroid hormone primarily obtained through sunlight exposure. It plays a crucial role in neuronal differentiation, axonal connectivity, dopamine ontogeny, and transcription control of genes [84]. The vitamin D receptor is extensively expressed in the brain [85], particularly in the hippocampus and prefrontal cortex, regions associated with learning and memory. Hypovitaminosis D during pregnancy has been strongly linked to an increased risk of ASD and cognitive impairment [86,87]. Furthermore, low vitamin D levels lead to greater susceptibility to antioxidant stress, in turn leading to abnormal immune responses and an elevated risk of developing chronic inflammatory conditions, with an increased risk of alteration in fetal neurodevelopment [88].

### 2.2. Obesity

Maternal obesity has gained pandemic proportions in both low- and high-income countries. Both maternal obesity and a high-fat diet (HFD) can impact fetal programming, predisposing the offspring to the development of adverse cardiometabolic and neurodevelopmental outcomes [89]. Animal models investigated the associations between maternal obesity and adverse neurodevelopmental and psychiatric outcomes in the offspring, revealing a reduction in the proliferation and maturation of stem-like cells in the ventricular layer surrounding the third ventricle, hypothalamic region, hippocampus, and cerebral cortex [89]. At the same time, epidemiological studies suggested an association between maternal obesity and unfavorable neurodevelopmental outcomes in human offspring [89].

Several studies supported the association between maternal obesity and cognitive deficits, ADHD, autism, and psychoses in the offspring [90]. Animal studies showed that maternal HFD is associated with significant changes in genes methylation involved in synaptic function, chromatin remodeling, and transcription regulation, which play a crucial role in the development of autism spectrum disorder [32]. Moreover, disorders within the mTOR and MAPK pathways have been observed in offspring exposed to maternal HFD, which can strongly increase the maternal inflammation state [33]. Alterations in these pathways are associated with autistic-like behavior in male mice (but not in females) [33]. This difference seems to be associated with the different exposure to androgens, which also influence the increased susceptibility of the male fetus to inflammation in utero, and the different activities of astrocytes and microglia [33]. Additionally, maternal obesity during pregnancy was reported to increase the odds of offspring cerebral palsy in a dose-dependent way [89]. Nevertheless, the association between the mother being overweight/obese and the offspring’s cognition is controversial, with some studies hypothesizing that the association may be confounded by genetic, socioeconomic, or postnatal factors [91]. Moreover, animal models found that maternal bariatric surgery led to significant changes in fetus DNA methylation without association with neurodevelopment delay in the offspring. Only one study reported more externalizing problems in children of mothers with preconception bariatric surgery compared to the control group [92]. A likely explanation is that the education level was significantly lower in mothers who underwent surgery. Other studies found no associations between bariatric surgery and offspring neurodevelopment [93,94]. However, further studies should evaluate the effects of bariatric surgery on fetal neurodevelopment [95].

### 2.3. Depression, Anxiety, and Stress

Depression and anxiety are the most common mental health symptoms during pregnancy, with prevalence rates varying depending on population characteristics, timing, and screening method. Approximately 14% to 54% of women are reported to experience anxiety symptoms during pregnancy within a low-risk, healthy, well-educated, and employed pregnant cohort [12]. Moreover, antenatal depression affects around 10–15% of pregnant individuals, with a significant number of women being affected by subsyndromal depressive symptoms, which are frequently overlooked [96,97,98]. Clinical evidence shows a chronic elevation of maternal glucocorticoids under stressful or depressive maternal conditions, accompanied by increased levels of pro-inflammatory cytokines associated with the increased risk of preterm birth (PTB) and neurodevelopmental pathologies [34]. Two prospective studies observed an association between antenatal maternal depression and alterations in neonatal microstructure of the right amygdala [10] and a decrease in cortical thickness [11]. Subsequently, antenatal depression is shown to be associated with disturbed or disorganized sleep [99], delays in acquiring language skills [100,101], and emotional and behavioral dysfunction [100,102,103,104] in neonates. Additionally, neonates born to mothers with depression during pregnancy have elevated levels of cortisol and catecholamines, resulting in more frequent crying and greater difficulty in being consoled compared to babies born to non-depressed mothers [97].

Studies on animal models subjected to prenatal stress showed alterations in the concentration of various neurotransmitters in the offspring, including dopamine and serotonin. These alterations appear to be associated with the development of idiopathic psychiatric disorders in adulthood, such as psychosis, mania, schizophrenia, and ADHD. Dopamine activity within the mesocorticolimbic pathways plays a pivotal role in cognition, emotion, positive reinforcement, food intake, and decision making [105]. While animal models confirmed the association between changes in dopaminergic mesolimbic and mesocortical pathways and psychiatric disorders, this link has not been directly assessed in human studies [105]. Additionally, mouse models demonstrated that a combination of maternal serotonin transporter (5-HTT) gene polymorphisms and prenatal stress increases the risk of ASD in the offspring. This condition may be associated with an imbalance between intra- and extra-cellular serotonin levels and reduced transporter binding availability. Indeed, serotonin is crucial for neurodevelopment during intrauterine life and subsequently regulates social behavior [35]. Furthermore, alterations in dopamine levels in the striatum have been observed in fetuses of pregnant dams with 5-HTT polymorphisms, which may be mitigated by DHA supplementation [36]. However, several human studies confirmed an increased risk of ASD in fetuses born after prenatal maternal stress exposure [106,107,108]. A recent cross-sectional study involving 459 mothers of children with autism showed an association between moderate family income and severity of ASD compared to high family income. A previous study reported that family income may act as a mediator in the progression of ASD [109].

Maternal anxiety and stress were associated with alterations in the fetal cortical gyrification index within the frontal and temporal lobes and reduced hippocampal volume during the late second and third trimesters of pregnancy [12]. According to this, a rat model showed an association between maternal stress and a significant reduction in hippocampal BDNF [34]. A recent trial involving pregnant women randomized via a mindfulness-based stress reduction program, reported that the left anterior cingulate lobe exhibited a larger volume and higher scores in regulating states of behavior on the Neonatal Behavioral Assessment Scale [8].

### 2.4. Smoking

Maternal smoking during pregnancy is one of the most prevalent environmental factors affecting fetal and neonatal growth. Nicotine and carbon monoxide exposure reduces oxygen availability, which is crucial for proper fetal development, leading to intrauterine chronic hypoxic status. Moreover, tobacco smoke contains numerous harmful chemicals, including tar (tobacco residue), which can cross the placenta and reach the developing fetus, potentially causing inflammation processes [110]. Indeed, chemicals in smoke may induce neuroinflammation by promoting oxidative stress, increasing levels of proinflammatory cytokines, and disrupting mitochondrial function. These damaging events may alter the immune functions of the fetal brain, making such offspring more vulnerable to brain insults [37,111].

Additionally, nicotine exposure is associated with alteration in DNA and histone methylation processes of the pivotal gene for neurodevelopment [112]. Animal models showed an association between maternal smoking and an altered expression of genes’ transcribing for Neural Cell Adhesion Molecule 1 and Neuroligin1, which modulate synapse development. Indeed, alterations in the expression of these genes appear to be implicated in offspring neuropsychiatric disorders [38,39]. Recently, several studies observed that smoking during pregnancy affects the formation of the fetal head shape by modulating the closure of cranial sutures [15,113]. Specifically, maternal smoking is associated with a statistically significant reduction in head circumference, without changes in biparietal diameter, left ventricular size, and cisterna magna [15]. Therefore, severe consequences for offspring are associated with maternal smoking [110]. Studies focusing on neonates born to mothers who smoke have reported an increased risk of neurodevelopmental disorders [114], such as ADHD, autism, schizophrenia, and behavioral issues [115,116,117].

### 2.5. Alcohol

According to the Global Status Report on Alcohol and Health 2018 by the World Health Organization (WHO), 42% of pregnancies are unplanned. At the same time, 65.5% of women of childbearing age in Europe consume alcohol, thereby increasing the risk of fetal exposure during the early weeks of pregnancy [118]. Fetal alcohol spectrum disorders (FASD) is an umbrella term that encompasses a range of adverse effects linked to alcohol exposure in utero. Fetal alcohol syndrome is a subset of FASD characterized by central nervous system damage, minor facial features, and growth alterations. The prevalence of FASD in Europe is 19.8 per 1000 children. Currently, there is no safe amount of alcohol consumption throughout the entire gestational period [119,120].

Prenatal alcohol exposure appears to affect fetal brain development by inducing uteroplacental insufficiency and hypoxic–ischemic lesions. This, in turn, leads to apoptosis in the developing neurons with a subsequent reduction in the overall number. Additionally, animal studies showed that ethanol metabolism to acetaldehyde and acetic acid induces neuroinflammation and generates ROS, leading to an increase in pro-inflammatory molecules, including IL-1α and CD24a mRNA, a suppression of anti-inflammatory PPAR-γ signaling, and induce programmed cell death. This subsequently results in crucial damage to the fetal brain [119,121], especially in the developing hippocampus [40].

Additionally, epigenetic mechanisms play a pivotal role. Alcohol exposure may lead to DNA methylation and histone modifications, such as acetylation [122]. These mechanisms are key epigenetic modifications that can alter chromatin structure and gene accessibility, thereby influencing gene expression. These epigenetic changes can be stable and may persist throughout an individual’s lifetime [41,123].

Furthermore, studies showed that cranial neural crest cells are the most susceptible to alcohol exposure, influencing the development of facial features in the fetus. Additionally, a reduction in cerebellar and brainstem volumes, as well as white matter, has been observed [42,124]. Human studies showed a reduction in brain size, particularly in the parietal and temporal lobes, and the corpus callosum. Moreover, MRI studies in children showed alterations in white matter microstructural integrity [13,14,119,125]. Among neonates exposed to prenatal alcohol, those with longer durations and higher doses showed poorer outcomes, including mild to severe behavioral and cognitive delays [126].

### 2.6. Air Pollution

The increasing emissions from rapidly growing modern industry and urbanization are extensively impacting on air quality. The WHO expressed concerns about the global impact of poor air quality on human health, based on studies showing the harmful effects of direct exposure to Particulate Matters (PMs) originating from fossil fuel, biomass burning, and traffic, as well as polycyclic aromatic hydrocarbons (PAH), which are a widespread environmental pollutant produced from the incomplete combustion of fossil fuels, tobacco, and other organic materials. PAHs may adhere to PM particles.

Currently, over 90% of the global population breathes air not meeting the WHO standards [127]. Additionally, prenatal exposure to air pollutants was shown to adversely affect fetal neurodevelopment, with male offspring being more susceptible to long-term cognitive and behavioral disorders [127]. Animal models showed that PM_2.5_ (PM with diameter of 2.5 μm or smaller) can cross the placenta and circulate in fetal blood, inducing oxidative stress and inflammatory responses in the growing fetus, thereby affecting fetal development [43,104,127,128,129]. Due to the immature or impaired blood–brain barrier function, PM can enter the fetal brain, activating inflammatory responses in astrocytes and microglia. Subsequently these cells release proinflammatory cytokines locally and activate inflammatory pathways, such as JNK and NF-κB, leading to the impairment of oligodendrocytes and damage to myelination in the white matter [127]. Additionally, PM can induce the production of reactive species of oxygen in the mother, which pass into fetal circulation and induce oxidative stress responses in the fetal brain, causing damage in brain regions such as the hippocampus [127,130]. Indeed, the exposure to PAH and PM_2.5._ during intrauterine life is associated with a reduction in weight and head circumference at birth [131]. Moreover, PAH is associated with a reduction in the white matter surface of the left hemisphere in childhood [16,131]. The alterations in brain developmental result in a lower Mental Development Index and have adverse effects on neonatal neurobehavior, manifesting as symptoms of anxiety, depression, and attention problems [132,133]. Particularly, maternal exposure to PM_2.5_ during pregnancy is associated with a higher ASD risk [134,135,136]. Animal models showed an association between prenatal exposure to PM in mice and increased anxiety and spatial memory dysfunction in adult male offspring, caused by altered expression of pro-inflammatory cytokines and N-methyl-D-aspartate receptor subunits in the hippocampus [44]. Similarly, prenatal exposure to diesel exhaust particles leads to a heightened susceptibility to behavioral deficits in adult offspring, particularly in males. This susceptibility is attributed to a significant upregulation of toll-like receptor 4 (TLR) expression and subsequent alterations in microglia activation [45].

### 2.7. Socioeconomic Status

Socioeconomic status (SES) can be determined by using variables such as health insurance status, education level, household income, material resources, and occupation [17,18,137]. A holistic approach that considers multiple indicators provides a more accurate and complete view of social condition. SES is one of the most significant factors linked to medical outcomes. Previous studies suggested that socially disadvantaged conditions are associated with pregnancy complications, including spontaneous miscarriage, preterm delivery, preeclampsia, eclampsia, and gestational diabetes [138,139,140]. A low SES is associated with more difficult access to healthcare, leading to poor fetal and maternal outcomes. Similarly, an unhealthy diet, lack of physical exercise, smoking, and alcohol consumption are more frequent in socially disadvantaged conditions, with significant consequences for both the fetus and the mother, as previously described.

Despite limited research, some studies observed that the prenatal maternal socioeconomic environment may affect infant neurodevelopment. Socially disadvantaged conditions were associated with a reduction in cortical and subcortical gray and white matter, as well as cortical folding in the first weeks of life [17]. Conversely, higher socioeconomic status is associated with increased volumes of developing fetal brain white matter, deep gray matter, cerebellum, and brainstem during pregnancy and decreased cortical gray matter volume [18,141]. Additionally, socioeconomic disadvantages are linked to increased stress levels, which, in turn, elevate maternal cortisol levels and result in smaller amygdala volumes [19]. Subsequently, a low maternal SES is associated with adverse outcomes in childhood neurodevelopment, impacting physiological and psychological health, cognitive development, educational attainment, and socio-emotional well-being [142].

### 2.8. Gut Microbiota

The gut microbiota refers to the entirety of microorganisms residing in the gastrointestinal tract of an individual, particularly the colon. This complex ecosystem consists of bacteria, viruses, fungi, and other microbes [143]. Environmental factors, along with nutritional status, diet, stress, infection, lifestyle, and antibiotic and antidepressant use, can influence the composition of maternal and fetal gut microbiota leading to a condition of dysbiosis. Dysbiosis is associated with a subsequent increase in inflammation and metabolic endotoxemia, leading to higher adverse risk for both the mother and the fetus [144,145]. Several studies observed a link between maternal dysbiosis and fetal brain development [143,146]. Two distinct theories were proposed to explain how the maternal intestinal microbiota could significantly affects the fetal side. One hypothesis suggests that microbes from the maternal site are translocated from the intestinal epithelium into the bloodstream and then delivered to the placenta. The second possible pathway involves the passage of microbiota-derived metabolites trans-placentally to the fetus [143].

Several animal and human studies observed that an alteration in the composition of maternal microbiota during pregnancy is associated with worse behavioral outcomes, primarily due to higher internalizing symptoms and autism-like behaviors [144,146]. Rodent studies demonstrated alterations in gene expression related to neurotransmission, neuroplasticity, metabolism, and morphology in both the hippocampus and thalamocortical neurodevelopment, linked to sensorimotor behavior and pain perception postnatally [144].

## 3. Inflammation and Altered Nutrient Supply: Models of Intrauterine Health Programming

**Figure 1:**
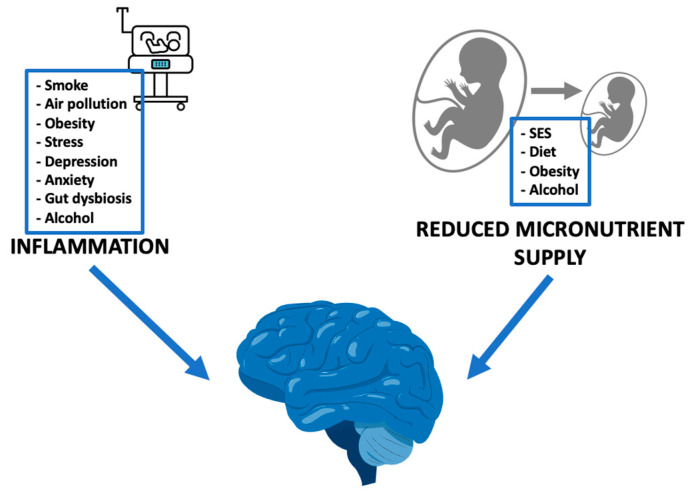
External stimuli, through inflammation and reduced micronutrient supply, impact on fetal neurodevelopment. SES: socioeconomic status.

A wealth of studies indicated an association between pathological pregnancy conditions, such as fetal growth restriction (FGR) and PTB, and neurodevelopmental derangements in the offspring. It is possible to delineate two different phenotypes that can lead to alterations in fetal programming and neurodevelopment: first, altered fetal nutrient supply and second, intrauterine inflammation (Figure 1), both implying placental involvements. FGR and PTB serve as paradigms, illustrating how these mechanisms lead to a substantial impact on fetal brain development.

### 3.1. Fetal Growth Restriction: Model of Reduced Nutrient Supply

FGR is a condition in which the fetus does not reach its genetic growth potential [147]. Approximately 60–70% of FGR cases are associated with placental dysfunction, leading to a reduced supply of nutrients and oxygen to the fetus, thereby inducing a state of chronic hypoxia and fetal undernutrition. In response, the fetus sends signals to the brainstem via carotid and central chemoreceptors, triggering an adaptative response known as brain sparing. This adaptation involves the redistribution of cerebral–placental blood flow, optimizing enhanced oxygenation to the essential organs, particularly the brain. Despite these mechanisms, FGR fetuses can experience significant consequences to fetal neurodevelopment, leading to long term adverse outcomes [148,149].

Indeed, intrauterine chronic hypoxia is associated with a reduction in cortical grey and white matter [150], accompanied by a larger depth of the Sylvian fissure. Areas more sensitive to hypoxia include the hippocampus, amygdala, basal ganglia, thalamus, and cortical areas [151]. Moreover, FGR fetuses may undergo increased stress, resulting in higher cortisol levels that can impact gyrification [152]. Following childbirth, a subsequent delay in both developmental and behavioral outcomes have been observed in the FGR offspring [149,151,152].

### 3.2. Preterm Born Children: Model of Intrauterine Inflammation

The incidence of PTB accounts for about 10% of live births globally, with a significant discrepancy between high- and low- income countries. Approximately 50% of preterm deliveries are linked to maternal inflammation or infection [153]. PTB is the leading cause of death in children under 5 years worldwide and is strongly associated with both short- and long-term morbidities in the offspring [154], including neurodevelopment impairments [153].

MRI studies conducted on preterm born children showed alterations in the axonal and neuronal development [155]. Injury to the developing white matter can result in multiple brain abnormalities, including interruptions in thalamocortical, corticothalamic, and cortico-cortical connections, as well as a decrease in cortical and deep nuclear grey matter volumes [155]. This results in different pathologies, from neurocognitive delay to cerebral palsy or periventricular leukomalacia.

The premature brain is vulnerable to injury for various reasons, including the immaturity of the blood–brain barrier, limited myelination, inability to produce anti-inflammatory cytokines, and deficiency in endogenous trophic factors, such as allopregnanolone, particularly in early gestational stages [153]. After intraventricular hemorrhage, which occurs in 20% to 40% of all preterm infants born weighting less than 1500 g [156], inflammation and infection play a pivotal role in brain injury and developmental abnormalities in preterm neonates [152,155]. Infectious and inflammatory processes lead to the activation of microglia and the production of chemokines and cytokines. In high concentrations, these molecules can damage oligodendrocytes and neurons. Additionally, the activation of microglia may contribute to an increase in free radical production and subsequent cell death [155,157,158]. Moreover, FGR fetuses that are born prematurely may show altered placental biomagnification of omega 3 fatty acids [159] and delayed cerebral maturation in association with brain sparing [160].

### 3.3. Inflammation

Inflammation influences the regulation of insulin, glucose, and leptin signaling in the developing brain, alters dopaminergic and serotonergic signaling, and impairs reward circuitry. These processes can lead to a decrease in the expression of brain-derived neurotrophic factor, resulting in the dysregulation of synaptic plasticity [89].

Obese pregnant women further represent a model of chronic low-grade systemic inflammation, attributed to the release of circulating pro-inflammatory cytokine levels (tumor necrosis factor-alpha (TNF-a), IL-1, IL-6, insulin, and leptin) and oxidative stress from adipose fat cells [145,161,162,163]. Furthermore, stress, depression, and anxiety are associated with an increase in maternal inflammatory status. The underlying reason is not well understood, but it appears to be linked to dysregulation in glucocorticoid production, resulting in the exacerbation of pro-inflammatory cytokine secretion, the alteration of maternal gastrointestinal microbiome, and a higher level of chronic stress [164]. Other conditions, such as maternal gut dysbiosis and air pollution, may contribute to an increase in maternal inflammation [128,165,166]. Particularly, air pollution and maternal immune activation appear to act together on the fetal brain [166], causing neuroinflammation and increasing the risk of neuropathology in offspring, including ASD, schizophrenia, bipolar disorder, major depressive disorder, epilepsy, and cerebral palsy [167].

Additionally, temporary conditions such as periodontal diseases may be more frequent in pregnant women. Smoking has an enormous influence on the development and progression of periodontal disease, impacting the gums and supporting structures of the teeth. Therefore, smoking and obesity can synergistically amplify the maternal inflammatory and oxidative status [113,168,169]. Several studies showed that maternal immune system activation leads to the release of proinflammatory cytokines. These cytokines cross the blood–brain barrier, leading to microglia activation, which, in turn, induces oxidative stress and mitochondrial dysfunction [167]. Oxidative stress is an imbalance between reactive oxygen species and antioxidants. Inflammation and oxidative stress form a self-perpetuating vicious cycle, resulting in downstream abnormalities in brain development and behavior [167]. Furthermore, both animal and human models showed how exposure to toxic, environmental, or occupational chemicals may lead to the production of maternal antibodies, likely as a response of the maternal immune system to external stimuli. In instances where the blood–brain barrier is compromised, these antibodies are facilitated to cross into the fetal brain, leading to additional impairment [170].

In conclusion, maternal conditions such as obesity, infection, and smoking, along with external stimuli like air pollution, act synergistically to play a crucial role in increasing the maternal inflammatory state [171].

## 4. Conclusions and Future Directions

The present review highlights the crucial role of external stimuli during pregnancy in influencing fetal growth and development. The intrauterine environment has an impact on fetal brain development, influencing long-term neurodevelopmental outcomes.

Recently, several studies investigated the alteration of the fetal brain in medical conditions such as FGR and PTB, showing that changes in nutrient supply and inflammation are associated with alterations in the morphometry of fetal brain regions and significant consequences for offspring. Similarly, unhealthy dietary patterns before or during pregnancy, often prevalent in obese women, or associated with anxiety disorder, depression, and low SES, can lead to a decrease in micronutrient supply. All these factors may lead to a reduction in total brain volume and impairment in brain structures. Moreover, air pollution, smoke, stress, depression, anxiety, and obesity increase the state of maternal inflammation, which is known to play a pivotal role in epigenetic mechanisms and, therefore, fetal neurodevelopment. Thus, the environment plays a crucial role in fetal development processes through epigenetic mechanisms modified by inflammation or alterations in nutrient supply. Identifying modifiable environmental risk factors should be mandatory for healthcare professionals and clinicians to reduce adverse effects. In fact, long-term outcomes impact not only individual health but also public healthcare. Therefore, in today’s world, prevention should be the focus for healthcare providers. To achieve this, further studies with longitudinal follow-up are necessary to understand the intricate interplay between the intrauterine environment, epigenetic modifications, fetal brain development, and offspring outcomes.

## Figures and Tables

**Table 1:** Detailed summary of the primary human studies on fetal neurodevelopment and maternal risk factors.

Author, Year	Type of Study	Investigated Variable	Population	Main Findings
A. Nakaki [8], 2023	Randomized clinical trial	Mediterranean Diet	90 fetal MRI	TBV is positively associated with walnut intake and biomarkers of olive oil consumption.
		Stress	90 fetal MRI	The stress reduction group has larger volume of left anterior cingulate lobe.
E. Ogundipe [9], 2018	Double-blind randomized placebo-controlled study	DHA	84 neonatal MRI	Males born from supplemented mothers (600 mg of DHA) showed larger volume in total brain, cortex, whole gray matter and corpus callosum compared to controls.
A. Rifkin-Graboi [10], 2013	Prospective observational study	Depression	157 neonatal MRI	Changes in microstructure of the right amygdala.
C. Lebel [11], 2016	Prospective observational study	Depression	52 children MRI	altered gray matter structure in children.
Y. Wu [12], 2020	Prospective cohort study	Anxiety	193 fetal MRI	Reduced fetal hippocampal volume, particularly in the left hippocampus. Maternal anxiety and stress are associated with changes in fetal cortical gyrification index of the frontal and temporal lobes.
S. W. Jacobson [13], 2017	Prospective observational study	Alcohol	32 children MRI	Smaller CC in infants with FAS diagnoses.
A. Roos [14], 2021	Prospective observational study	Alcohol	83 children MRI	Alterations in white matter microstructural integrity in children with PAE.
C. Çetin [15], 2023	Prospective observational study	Smoke	250 fetal US	Significant reduction in second trimester measurement compared to non-exposed fetuses.
B. S. Peterson [16], 2015	Cross-sectional study	PAH exposure	40 children MRI	Reduction in the surface area of WM, predominantly confined to the left hemisphere of the brain.
R. L. Triplett [17], 2022	Prospective longitudinal cohort study	Prenatal social disadvantage	280 neonatal MRI	Reductions in WM, cortical gray matter, and subcortical gray matter volumes and cortical folding.
Y. Lu [18], 2021	Prospective longitudinal cohort study	Socioeconomic status	144 fetal MRI	Higher socioeconomic status is associated with: - increased volumes of the developing fetal brain WM, DGM, cerebellum, and brainstem during pregnancy - decreased CGM volume.
M. P. Herzberg [19], 2023	Longitudinal observational study	Socioeconomic status	241 neonatal MRI	Socioeconomic disadvantages are associated with higher cortisol level and smaller amygdala volumes.

MRI: magnetic resonance imaging, TBV: total brain volume, DHA: docosahexaenoic acid, CC: corpus callosum, FAS: fetal alcohol syndrome, PAE: prenatal alcohol exposure, US: ultrasound, HC: head circumference, BPD: biparietal diameter, LV: lateral ventricular, CM: cisterna magna, PAH: polycyclic aromatic hydrocarbon, WM: white matter, DGM: deep gray matter, and CGM: cortical gray matter.

**Table 2:** Detailed summary of the animal studies on fetal neurodevelopment and maternal risk factors.

Author, Year	Model	Investigated Variable	Main Findings
Belluscio L [20], 2014	Mouse	Low protein diet	Delays in the surface righting reflex and negative geotaxis response.
Fragoso J [21], 2021	Rat	Low protein diet	Reduction in the expression of BDNF in the hypothalamus and motor cortex.
Li C [22], 2017	Baboon	Undernourished	Suppression of neurotrophic factors, dysregulated cell proliferation, and impaired glial maturation and neural synthesis.
Rajarethnem HT [23], 2017	Rat	DHA supplementation	Significant increase in the number of hippocampal neural cells.
Fedorova I [24], 2009	Rat	DHA	Low brain DHA is associated with a deficit in spatial reversal learning that could be related to changes in dopamine transmission in critical brain circuits.
Liu D [25], 2010	Rat	Iodine	Hypothyroidism is associated with: -decrease in BDNF mRNA expression in the hippocampus -long-term memory deficits of pups.
Zhan Y [26], 2015	Rat	Iodine	Hypothyroidism is associated with: -decreased activation of the CREB signaling pathway -impairments of cognitive function.
Tran P [27], 2015	Rat	Iron Deficiency	Significant epigenetic modifications lead to long-term repression of BDNF.
Lien Y [28], 2019	Rat	Iron Deficiency	Changes in DNA methylation in neural gene.
Barks A [29], 2022	Rat	Iron Deficiency	TET/DNA hydroxymethylation system is disrupted in a brain region-specific manner.
Zhan X [30], 2012	Rat	Folic Acid deficiency	Reduces neuronal cell proliferation by impairing mitosis and increases apoptosis.
Araki R [31], 2021	Mouse	Low folate	Influences DNA and histone methylation, leading to the downregulation of neural gene expression and impaired fetal neurogenesis.
Gawlinska K [32], 2021	Rat	High-fat diet	Changes in genes methylation, which are involved in synaptic function, chromatin remodeling and transcription regulation.
Gawlinska K [33], 2021	Rat	High-fat diet	Alteration in mTOR and MAPK pathways are associated with autistic-like behavior.
Czarzasta K [34], 2019	Rat	Depression	Altered levels of BDNF in the cerebellum and hippocampus have been associated with neurodevelopmental and behavioral delays in offspring.
Jones J [35], 2010	Mouse	Prenatal stress	Combination of 5-HTT polymorphisms and prenatal stress increases the risk of ASD.
Matsui F [36], 2018	Mouse	Prenatal stress	In mice with 5-HTT polymorphisms, dopamine levels increase significantly in the striatum. DHA supplementation reduces dopamine levels.
Chan Y [37], 2016	Mouse	Smoke exposure	Smoke increases markers of hypoxia, oxidative stress and inflammation in neural cells, which may render dams and their offspring vulnerable to additional brain insults.
Jung S-Y [38], 2010	Rat	Smoke exposure	Neuroligin-1 can modulate synaptic plasticity in the amygdala circuits of adult animals, likely by regulating the abundance of postsynaptic NMDA receptors.
Xiao M-F [39], 2009	Mouse	Smoke exposure	NCAM is a modulator of the dopaminergic system, playing an important role in the etiology of psychiatric disorders.
Niedzwiedz-Massey V-M [40], 2021	Mouse	Alcohol	Ethanol induces neuroinflammation, reduces the expression of molecules associated with mature oligodendrocytes, and leads to a decrease in genes expressed in oligodendrocyte progenitor cells.
Cantacorps L [41], 2019	Mouse	Alcohol	Alcohol induces persistent epigenetic modifications, leading to long-term cognitive and behavioral impairments.
Milbocker K [42], 2022	Rat	Alcohol	Alcohol leads to alterations in corpus callosum development and in myelination process.
Cui J [43], 2019	Mouse	Air pollution	Activation of the dopamine pathway with the inhibition of glycine pathway can lead to locomotor hyperactivities.
Ehsanifar M [44], 2019	Mouse	Air pollution	Alteration in the expression of pro-inflammatory cytokines and N-methyl-D-aspartate receptor subunits in the hippocampus, leads to increased anxiety and spatial memory dysfunction in offspring.
Nicolas Z M [45], 2017	Mouse	Air pollution	Prenatal exposure to diesel exhaust particles leads to a significant upregulation of TLR4 expression and alterations in microglia activation, resulting in a higher vulnerability to behavioral deficits in male adult offspring.

BDNF: brain-derived neurotrophic factor, DHA: docosahexaenoic acid, CREB: cAMP response element-binding protein, TET/DNA: ten eleven translocation/DNA, mTOR: mechanistic target of rapamycin, MAPK: mitogen-activated protein kinase, 5-HTT: serotonin transporter, ASD: autism spectrum disorder, NMDA: N-methyl-D-aspartate receptor, NCAM: neural cell adhesion molecule, and TLR: toll like receptor.

## Data Availability

No new data were created. All the data reported in the paper are collected from published scientific papers.
